# Hypertension Experiences Affecting Recovery from Delivery (HEARD): a mixed-methods interview study of postpartum people affected by hypertensive disorders of pregnancy

**DOI:** 10.1186/s12884-025-08013-0

**Published:** 2025-09-30

**Authors:** Hana G Murphy, Angelina Noll, Elizabeth Langen, Ashley Hesson

**Affiliations:** 1 Department of Obstetrics/Gynecology, Kaiser Permanente San Francisco, San Francisco, CA USA; 2 University of Michigan Medical School, Ann Arbor, MI USA; 3 Department of Obstetrics & Gynecology, Division of Maternal Fetal Medicine, University of Michigan, Ann Arbor, MI USA

**Keywords:** Birth trauma, Hypertensive disorders of pregnancy, Pre-eclampsia, HELLP, Pregnancy, Prenatal care, Natural language processing

## Abstract

**Background:**

Postpartum care for hypertensive disorders of pregnancy (HDP) varies in terms of content, quality, and duration. People postpartum from pregnancies complicated by an HDP are known to have a variety of physical and emotional sequelae both from the HDP itself and associated complications (e.g., prematurity, traumatic birth). This study interviewed a geographically and socially diverse sample of recently postpartum people who had HDPs to better understand the needs of this population.

**Methods:**

Semi-structured interviews were conducted with participants recruited from social media. Both qualitative and quantitative methods were used to characterize the resulting transcripts. Transcripts were reviewed for patient-reported obstetric history and variables pertaining to intrapartum and postpartum care (e.g., gestational age at delivery, delivery mode). Birth experience was then categorized as positive, neutral, or negative by reviewer assessment. Sentiment analysis was performed to objectively identify elements of participants’ transcribed speech with emotive content. The emotive content of positive and negatively framed birth experiences was then qualitatively reviewed by thematic analysis.

**Results:**

67 interviews were analyzed from individuals affected by the entire spectrum of HDPs: 38.8% (*N* = 26) had gestational hypertension, 56.7% (*N* = 38) had a form of preeclampsia, and 4.5% (*N* = 3) had eclampsia or HELLP. Among those with negative birth experiences, fear was the dominant negative emotion identified in the sentiment analysis. This was driven by mentions of “anxiety” in reference to both the birth process and blood pressure management. Trust was the characteristic positive emotion, largely expressed by uses of the word “helpful”. Participants used “helpful” to identify services such as peer support communities that would have been beneficial to them or counseling they would have liked to receive about HDPs or their long-term effects. They identified lack of information and education about HDPs as a key gap in current practice.

**Conclusions:**

People postpartum from HDPs who described negative experiences felt that more information about their diagnosis and its management from their obstetric providers would have improved their experience along with postpartum support from people who had similar lived experiences. These insights point to practical interventions that can be undertaken both nationally and institutionally to enhance postpartum HDP care.

**Supplementary Information:**

The online version contains supplementary material available at 10.1186/s12884-025-08013-0.

## Introduction

Hypertensive disorders in pregnancy (HDP) – gestational hypertension, preeclampsia, eclampsia, HELLP Syndrome, and superimposed preeclampsia – are common pregnancy complications in the United States (US) that contribute substantially to pregnancy-related mortality and morbidity [1]. The physical and emotional sequelae of these disease processes extend far beyond the immediate peripartum period, and disproportionately affect Black and non-Hispanic White pregnant people, as well as those living in lower income areas [2]. Those with a history of an HDP have elevated cardiovascular risk that manifests in a higher lifetime incidence of hypertension, myocardial infarction, stroke, and dementia [3, 4]. Patients also experience greater rates of hospital readmission [5] which has the potential to impact maternal-infant bonding, breastfeeding, and mental health symptoms. They have a higher rate of mood disorders [6] and posttraumatic stress disorders stemming from their peripartum experiences [7] which can have lifelong effects on reproductive decision-making, interpersonal relationships, and interactions with the healthcare system.

The present U.S. postpartum care model for HDP varies by practice though often consists of regular blood pressure checks either virtual or in-person, and a single comprehensive postpartum visit four to six weeks following delivery [8]. Counseling on the long-term health risks of HDPs has been shown to be infrequent and of varying quality when it does occur [9]. Even when substantive counseling on risks is provided, no specific interventions for risk reduction exist. Lifestyle interventions such as diet and exercise are known to lower cardiovascular risk and improve mood [10], but there are no graded, supervised exercise programs or proven nutritional interventions offered at a national level to support patients in their physical and mental recovery. This is in stark contrast to the resources recommended to those with cardiovascular risk factors unrelated to a recent pregnancy, where cardiac rehabilitation programs have been shown to improve quality of life, reduce modifiable risk factors for cardiovascular disease, and prevent hospital readmission [11]. Attempts at clinic-based programs for physical and mental support after an HDP have faced poor attendance [12], suggesting that the needs of this population may be different than those of people recovering from a cardiovascular risk modifying events outside of the postpartum period.

The lack of a patient-driven, generalizable program to support patients’ recovery from an HDP is due at least in part to an incomplete understanding of what patients want from their recovery process. Existing research on the patient experience in postpartum recovery from an HDP has either been exclusively survey based or limited in generalizability. Multiple surveys have been fielded regarding blood pressure monitoring programs [13], but these offer limited insight to patients’ preferences for interventions. Small-scale interview studies provide more nuance regarding patients’ birth and postpartum experiences with HDPs, but their small sample sizes and limited geographic sampling make it difficult to create a larger-scale program based on their identified needs [14, 15].

To address the knowledge gaps surrounding the peripartum needs of those recovering from the physical, mental, and emotional aftermath of an HDP-affected birth, we interviewed postpartum people from the U.S. and Canada regarding their perspectives on improving post-HDP care. Our aim was to better understand patients’ birth and recovery experiences while also identifying barriers and opportunities for intervention. Given the significant morbidity and mortality associated with HDP, we aimed to identify tangible intervention points and support measures that both decrease HDP-associated morbidity in the short and long-term, as well as improve the HDP patient’s peripartum experience.

## Materials & methods

### Study participants

Participants were recruited via social media, specifically Facebook support groups for individuals who experienced HDP. Invitations to participate were also distributed to patients indirectly through national physician Facebook groups. Additional recruiting was achieved through physical flyers postings at our tertiary care center.

Interested individuals completed a screening questionnaire to determine study eligibility and provide demographic information. To maintain patient privacy and encourage patient comfort in disclosing personal experiences, this survey data was not linked to subsequent interview data though this meant we could not analyze responses by demographic groups. Potential participants had to meet the following inclusion criteria: age 18 or over, a diagnosis of a hypertensive disorder of pregnancy (i.e. gestational hypertension, pre-eclampsia, eclampsia, HELLP syndrome), and delivered within the previous 18 months. If the potential participant answered affirmatively to the screen in questions, they were asked questions about their basic demographics (self-identified race, age at time of delivery), details about their birth (gestational age at delivery, mode of delivery), and their socioeconomic status. The questionnaire can be found in Appendix A. To schedule an interview, the potential participant also needed to have an IP address locating them to the U.S. or Canada.

Participants who completed an interview were compensated for their time with a monetary gift card. Given the anonymous nature of the research and the potential for abuse of the compensation system, a screening algorithm based on questionnaire responses was used to exclude would-be participants whose answers or method of completing the screener were suspicious for fraud (e.g., a completion time less than two standard deviations under the mean, answers that were medically implausible, or exactly duplicated answers). Potential participants who were screened out based on their responses were not included in the demographic characterization of the sample. Eligible participants were invited to participate in a 45-min interview led by a member of our internal research team, or a trained moderator employed by a third-party qualitative research company, Verilogue Inc.

Verilogue, Inc. facilitated interview scheduling such that interviewers and interviewees were paired onto a virtual meeting platform invite. Email addresses were used to send the invite but were stored separately from the resulting data and were not accessible to interviewers. Participants were encouraged to display/use a pseudonym for the virtual meeting platform. They could elect to turn their video on or off per their preference. Interviewers from Verilogue, Inc were trained as part of the research team to ensure consistency. The interviewer team was all women with qualitative research experience, most of whom were White-presenting. Interviews were conducted from November 2022-January 2023. The University of Michigan Institutional Research Board determined this research to be exempt from review given the lack of personally identifiable data collected (HUM00216335).

### Materials

Interviews followed a semi-structured discussion guide that included the following topics: obstetric history and pregnancy course specific to this delivery, subjective birth experience, interactions with members of the health care team, postpartum course including complications, utilization of physical, mental, emotional resources in the postpartum period, and feelings of overall satisfaction with counseling and postpartum experience, in addition to what types of support would have been desired. The interview guide can be found in Appendix B.

Transcripts were generated by trained transcriptionists contracted by Verilogue, Inc. Any incidentally disclosed, potentially identifying information was blinded during the transcription process prior to analysis. The de-identified transcripts were provided to the research team in digital format.

### Analytic approach

Interview transcripts were primarily reviewed by one member of the research team, who extracted discrete categorical data, detailed in Supplemental Table 1, related to patient-reported birth and postpartum experience (e.g., first birth, preterm versus term birth, type of obstetrical provider). Reviewers subjectively assessed participants’ characterization of their births as “positive”, “neutral”, or “negative”. This was done based on reviewers’ intuition with respect to the speaker’s intended framing, without any prescriptive guidelines. When a determination could not be made by a single reader, transcripts or segments were reviewed by the research team until consensus was reached [16].

Variables characterizing participants’ birth and postpartum experiences as well as their preferences for postpartum recovery care were recorded by each reviewer (e.g., desired postpartum exercise and diet support, preferred source of patient education) and further outlined in Supplemental Table 1. Descriptive statistics were applied where appropriate.

Sentiment analysis was used to objectively quantify patterns in words and phrases with emotive or relational valence in interviewee-generated content (see Supplemental Table 2 for definitions and examples). The goal of this analysis was to identify areas of emphasis in interviewees’ transcribed speech, where speakers were attributing personal value (either positive or negative) to the topic under discussion. In behavioral research, sentiment analysis enables efficient analysis of verbal behavior to allow the study of emotional content in naturally occurring language [17–21]. To apply this technique, the interviewee transcribed speech was isolated from each interview. Comments made by the participants were classified as either birth or postpartum talk by a member of the research team. Automated filters were applied for function words and fillers (e.g., “uh”, “um”). Lexicons, collections of words representing a common theme or concept, were then searched within the interviewee’s transcribed text.

Two lexicons were used to classify words and phrases in transcribed interviewee speech: 1. Birth trauma lexicon (BTL): A custom lexicon was built from an independent training dataset of transcribed speech from recently pregnant people recalling their self-identified traumatic births [7] and 2. The NRC Sentiment and Emotion Lexicons: a validated set of emotive terms representing joy, trust, anticipation, surprise, sadness, disgust, fear, and anger [22]. The Birth trauma lexicon was built based on common n-grams (words and co-occurring combinations of words) in the training set and manually reviewed by a trained linguist (AH). The NRC Sentiment lexicon was reviewed for terms that may have a priori inapplicability to the context under study or potential for contextual misconstrual; said terms were removed prior to analysis. The BTL and modified NRC lexicons used for analysis included a combined total of 14,000 terms.

Both lexicons were applied using a search algorithm to the pre-processed interviewee transcribed speech. Words and phrases matching items from each lexicon were assigned polarity based on co-occurring negation terms to avoid misassignment of a given sentiment sub-lexicon; for example, “not mad” would be flagged as having negative polarity and removed from the set of term matches for *anger* (sentiment sub-lexicons are *italicized* to disambiguate sentiments from matched words). The remaining word and phrase matches were classified according to the sentiment term they matched: *trauma* (for BTL matches), *joy, trust, anticipation, surprise, sadness, disgust, fear,* and *anger.* Percentages of total words matched out of all the interviewee pre-processed transcribed speech were calculated for each term. Individual words were then scored with respect to their matched term (e.g., the word “mad” in transcribed speech matching the lexicon term *anger*). Scores were calculated as a proportion of within-term matches accounted for by that word (e.g., the extent to which *anger* is expressed using the word “mad”). The matches were further grouped into negative and positive birth experience and postpartum experience talk respectively using manual assignments made prior to term identification.

Though sentiment analysis has the benefit of objective pattern recognition and quantification, its interpretive value is limited without consideration of the context in which emotive terms occur. With the goal of identifying current care gaps and needs, we manually reviewed a subset of matches for unambiguously negative emotions of *trauma, anger, fear*, and *sadness* in birth and postpartum experiences. Where more than ten examples existed of a given term, a random number generator was used to select ten turns-at-talk, abbreviated as “turns”, in which the term was used for qualitative review and thematic analysis. Themes were derived from qualitative review of interviewee turns containing negative emotion terms. Tone, “negative”, “positive”, or “neutral” was also assigned. Each usage instance for the terms analyzed was assigned a theme and tone by two independent, blinded research team members. A third team member then reviewed the assignments to create a shared set of themes applied consistently across the negative emotion talk. Where disagreements existed on tone of the turn containing the negative emotive turn, majority consensus was achieved via the third reviewer.

## Results

Out of 188 respondents to the screening questionnaire, a total of 67 interviews were completed, transcribed, and analyzed after confirmed participant eligibility and excluding respondents flagged by the screening algorithm. The 121 respondents who were not interviewed were either screened out or did not complete the scheduling process.

### Sample characterization

Table 1 summarizes the demographic data from the participants who completed interviews. The majority of interviewees self-identified as White or Caucasian (*N* = 51, 76.1%). A variety of hypertensive diagnoses were represented: 38.8% (*N* = 26) had gestational hypertension, 19.4% (*N* = 13) had chronic hypertension with superimposed preeclampsia, 37.3% (*N* = 25) had preeclampsia either with or without severe features (including superimposed, 56.7%, *N* = 38), and 4.5% (*N* = 3) had eclampsia or HELLP. The majority were employed, privately insured, securely housed, and had at least a high school education.

**Table 1:** Demographics of the *N* = 67 participants who completed the interview process

	***N*** (%)
HDP diagnosis	
Chronic hypertension with superimposed preeclampsia	13 (19.4%)
Gestational hypertension	26 (38.8%)
Preeclampsia (with and without severe features)	25 (37.3%)
Eclampsia	1 (1.5%)
HELLP	2 (3.0%)
Race	
White	51 (76.1%)
Black or African American	12 (17.9%)
Hispanic or Latino	4 (6.0%)
Concern for Housing Instability	
Yes	7 (10.4%)
No	59 (88.1%)
Highest level Education	
High school diploma or GED	9 (13.4%)
More than high school	58 (86.6%)
Employed	
Full-time work	46 (68.7%)
Part-time or temporary work	13 (19.4%)
Otherwise unemployed but not seeking work (e.g. student, retired, disabled, unpaid primary caregiver)	6 (9.0%)
Unemployed	2 (3.0%)
Insurance Status	
Private Insurance	37 (55.2%)
Insurance (not CHIP)	12 (17.9%)
CHIP Medicaid Medicare	9 (13.4%)
Other Public	6 (9.0%)
None/Uninsured Medicaid	3 (4.5%)

The average age of participants at time of interview was 30.9 years (SD = 3.78). On average patients were 7.6 months postpartum (SD = 5.39) from their HDP-complicated pregnancy. Mean gestational age at HDP diagnosis was 36.8 weeks (SD = 2.02).

The majority of our sample were nulliparous individuals who delivered term infants via induction labor; 58.2% (*N* = 39) of participants birthed vaginally and 41.8% (N = 28) delivered via cesarean birth. Over half of patients reported receiving magnesium and a quarter required NICU care for their infants, see Table 2. A significant proportion experienced prolonged postpartum hospitalizations (> 72 h), often due to the need for inpatient blood pressure management (44.8%, *N* = 30).

**Table 2:** Intrapartum care and outcomes for the *N* = 67 participants who completed the interview process. All outcomes are participant reported

	**Yes**, *N * (%)	**No**, *N* (%)	**Unknown**, *N* (%)
Preterm (vs. Term) birth	13 19.4%	54 80.6%	0
Vaginal (vs. Cesarean) birth	39 58.2%	28 41.8%	0
Induced labor	50 74.6%	17 5.4%	0
Labored (including spontaneous and induced)	56 83.6%	11 16.4%	0
Received Magnesium	34 50.8%	31 46.3%	
Infant admitted to NICU	17 25.4%	50 74.6%	0
Maternal hospital stay > 72 h postpartum	30 44.8%	35 52.2%	23.0%
Nulliparous	52 77.6%	15 22.4%	0

Regarding specific outcomes related to management of HDP, 46.3% of patients required outpatient medication for blood pressure control and 10.5% went on to establish care with a cardiologist. The majority received counseling on home blood pressure monitoring (64.2%) but only 6.0% were counseled on the lifelong cardiovascular risks of an HDP diagnosis. A substantial proportion of patients were left unsatisfied by blood pressure counseling and management, 38.8% and 52.2% respectively. See Table 3 for a summary of hypertensive management and care.

**Table 3:** HDP care and outcomes for the *N* = 67 participants who completed the interview process. All outcomes are participant reported

	**Yes**, *N* (%)	**No**, *N* (%)	**Unknown**, *N* (%)
Experienced postpartum blood pressure complications	24	42	1
	35.8%	62.7%	1.5%
Experienced challenges with home blood pressure cuff	13	45	9
	19.4%	67.2%	13.4%
Discharged on hypertension medication	31	20	16
	46.3%	29.9%	23.9%
Received anticipatory guidance for home blood pressure management at discharge	43	15	8
	64.2%	22.4%	11.9%
Established care with cardiologist	7	39	21
	10.5%	58.2%	31.3%
Counseled on lifelong risks of HDP diagnosis	4	33	30
	6.0%	49.3%	44.8%
Satisfied with blood pressure monitoring anticipatory guidance	27	26	7
	40.3%	38.8%	10.5%
Satisfied with follow up for blood pressure management	25	35	6
	37.3%	52.2%	9.0%

### Birth experiences with hypertensive disorders

**Fig. 1:**
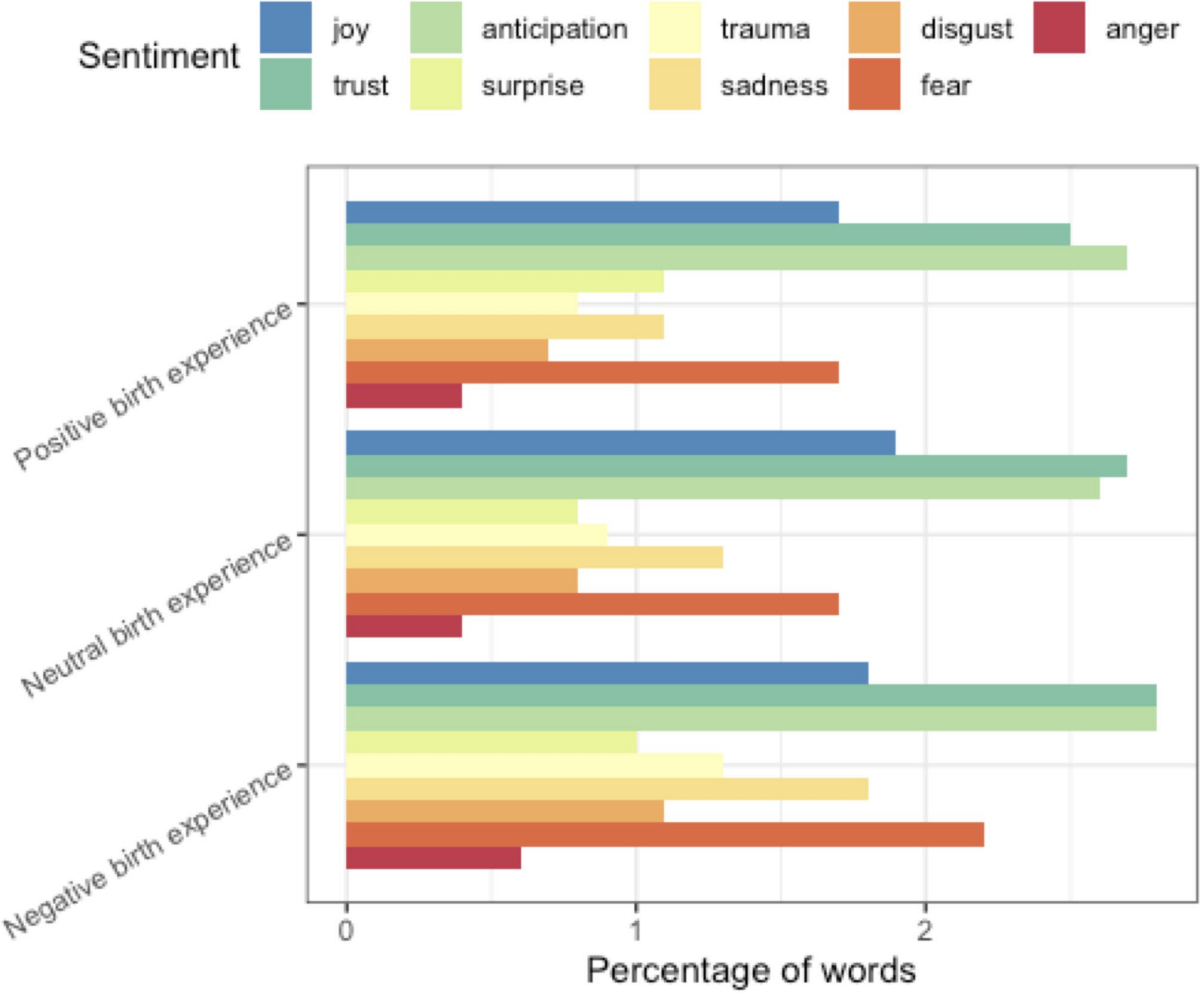
Quantitatively derived sentiments associated with positively, neutrally, and negatively framed birth experiences. Each sentiment sub-lexicon is plotted as a percentage of matched terms out of the total number of words categorized as negative, neutral, or positive birth experience talk respectively

Nearly a quarter of participants (23.9%, *N* = 16) had a positively framed birth experience, where 40.3% (*N* = 27) had a negatively framed one (35.8%, *N* = 24 were classified as “neutral). Negative birth experiences had disproportionate representation of words matching the *fear* sub-lexicon. *Anger, trauma,* and *sadness* were also over-represented in interviewee speech (see Fig. 1). *Trust* and *anticipation* were the most common sentiments across the three categories of birth experience, collectively representing over 4% of content words produced by interviewees discussing their birth experience.

**Fig. 2:**
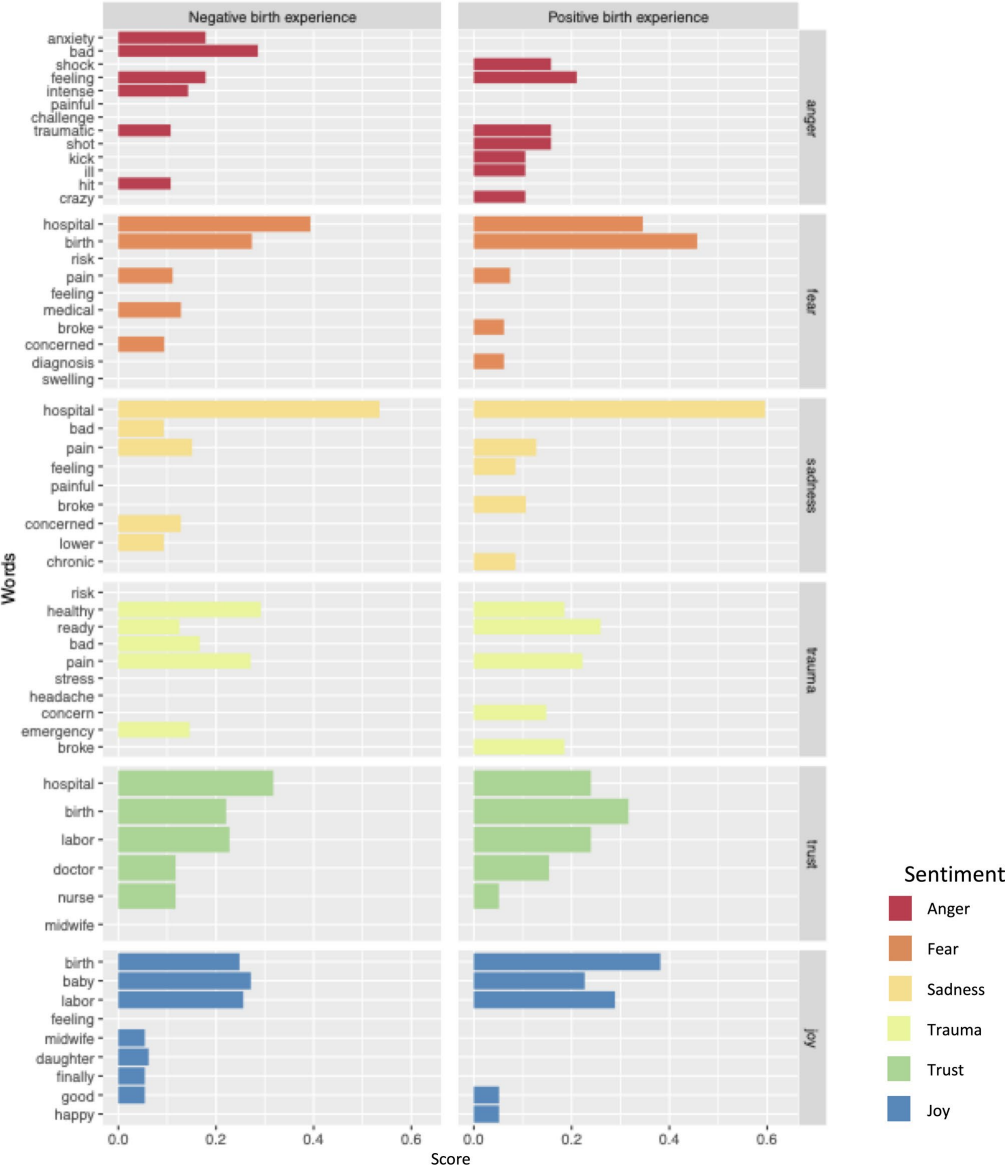
Matched terms for representative sentiment sub-lexicons in positively and negatively framed birth experiences. Each matched term’s score is a percentage of the terms matched within that sentiment that matched with respect to the given term

The individual words from each sub-lexicon that matched within interviewee birth experience speech are given in Fig. 2. For *trust* and *joy*, similar terms were represented at comparable scoring for the positively, neutrally, and negatively framed births. Use of the words “anxiety”, “intense”, and “emergency” distinguished the negatively framed birth experiences from the neutral or positive ones in the sub-lexicons for *anger, fear, trauma,* and *sadness*.

Participants who had negatively framed birth experiences perceived interrelations between their experience of anxiety and their blood pressure, which they expressed in the form of narratives describing their negative experiences with blood pressure monitoring:

“I have, I struggled with anxiety and I was very anxious throughout my whole pregnancy and I was pretty convinced for a lot of it that it was mostly, like, anxiety that was triggering it. And then, cause I would, like, panic when I got my blood pressure taken.”

Other mentions of “anxiety” referred to the birth process in general. Likewise, use of “intense” was not specific to having an HDP. Instead, it was used in descriptions of symptoms and the experience of being in labor:

“It didn't feel like a headache, it just felt like this really intense pins and needles tingling sensation but really not a headache but so that was a little bit confusing that I was having trouble explaining that to him.”

Uses of “emergency” were almost exclusively in co-occurrence with “c-section”:

“I had full fledged HELLP syndrome. Um, and so my platelets were at the point where, um, if I, if they didn't do an emergency C section, I, I wouldn't have been able to have any sort of, you know, but my platelets were low enough that I would've needed a transfusion, um, if I hadn't delivered right then.”

None of the *N* = 16 positively framed birth experiences were cesarean births; they were exclusively vaginal births. The vaginal birth rate was 48.1% (*N* = 13/27) in the negatively framed birth experiences and 62.5% (*N* = 15/24) in the neutrally framed birth experience group. For this reason uses of “emergent” cannot be directly compared across positively and negatively framed birth experiences.

Positive birth experiences have fewer mentions of “concern” and “anxiety”, with a relatively high proportion of “doctor” mentions. Talk matching the sub-lexicon of “trauma” is less common in positively framed birth experience talk than in neutral or negative birth experience talk.

### Postpartum experiences with hypertensive disorders

**Fig. 3:**
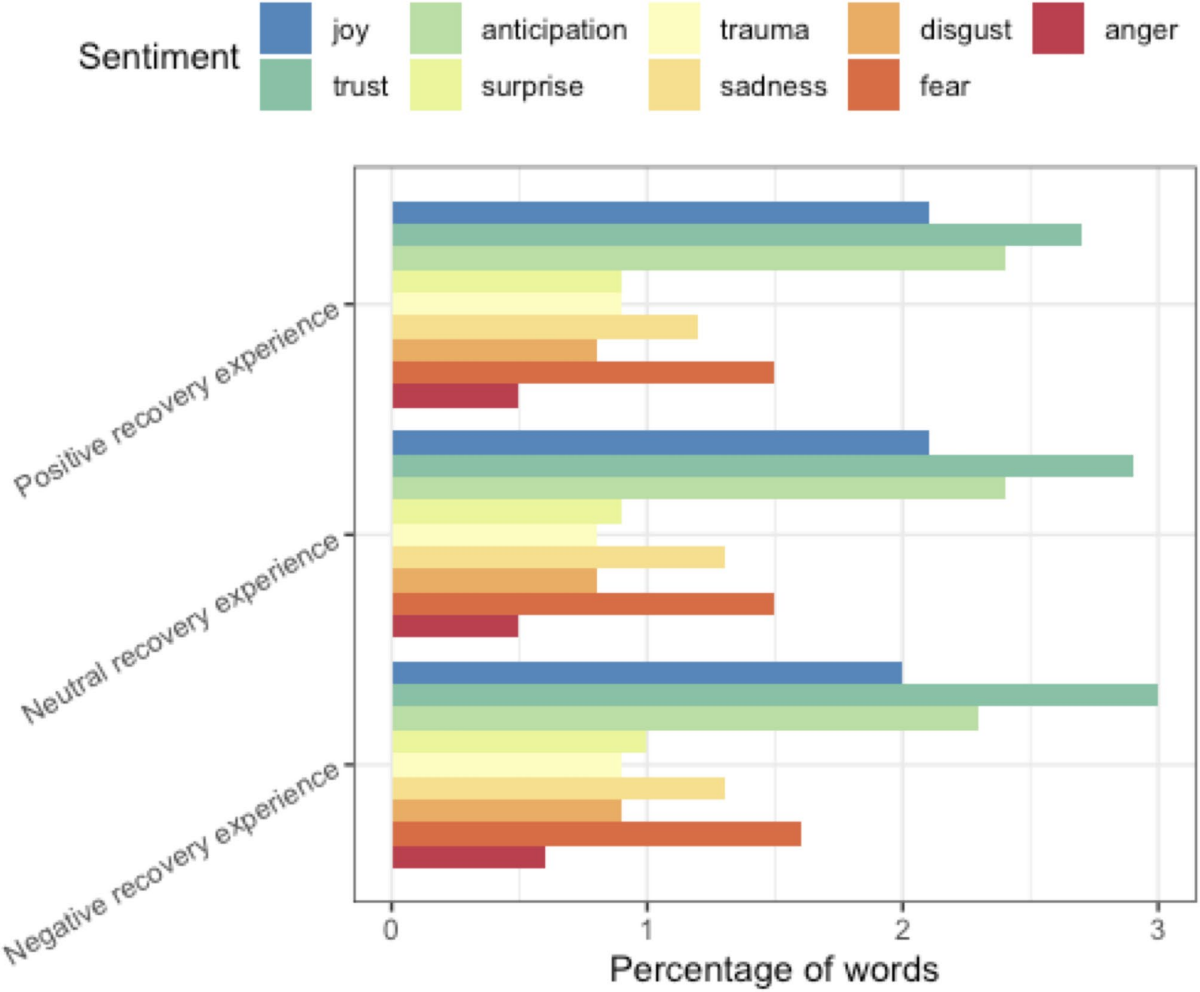
Quantitatively derived sentiments associated with positively, neutrally, and negatively framed postpartum recovery experiences. Each sentiment sub-lexicon is plotted as a percentage of matched terms out of the total number of words categorized as negative, neutral, or positive postpartum recovery experience talk respectively

Researchers classified a similar proportion of interviewees as having positively framed postpartum experiences (25.4%, *N* = 17). Approximately one third of interviewees (32.8%, *N* = 22) had negatively framed postpartum experiences and 41.9% (*N* = 28) had postpartum experiences that were classified as neutral. Unlike in their discussions about birth experiences, interviewees who had negatively framed postpartum experiences had comparable proportions of negative emotion sub-lexicons (*anger, fear, sadness, trauma, disgust*) identified in their speech. Slightly higher proportions of *trust* terms were seen in the negatively framed versus positively framed birth experience interviewee speech (see Fig. 3).

**Fig. 4:**
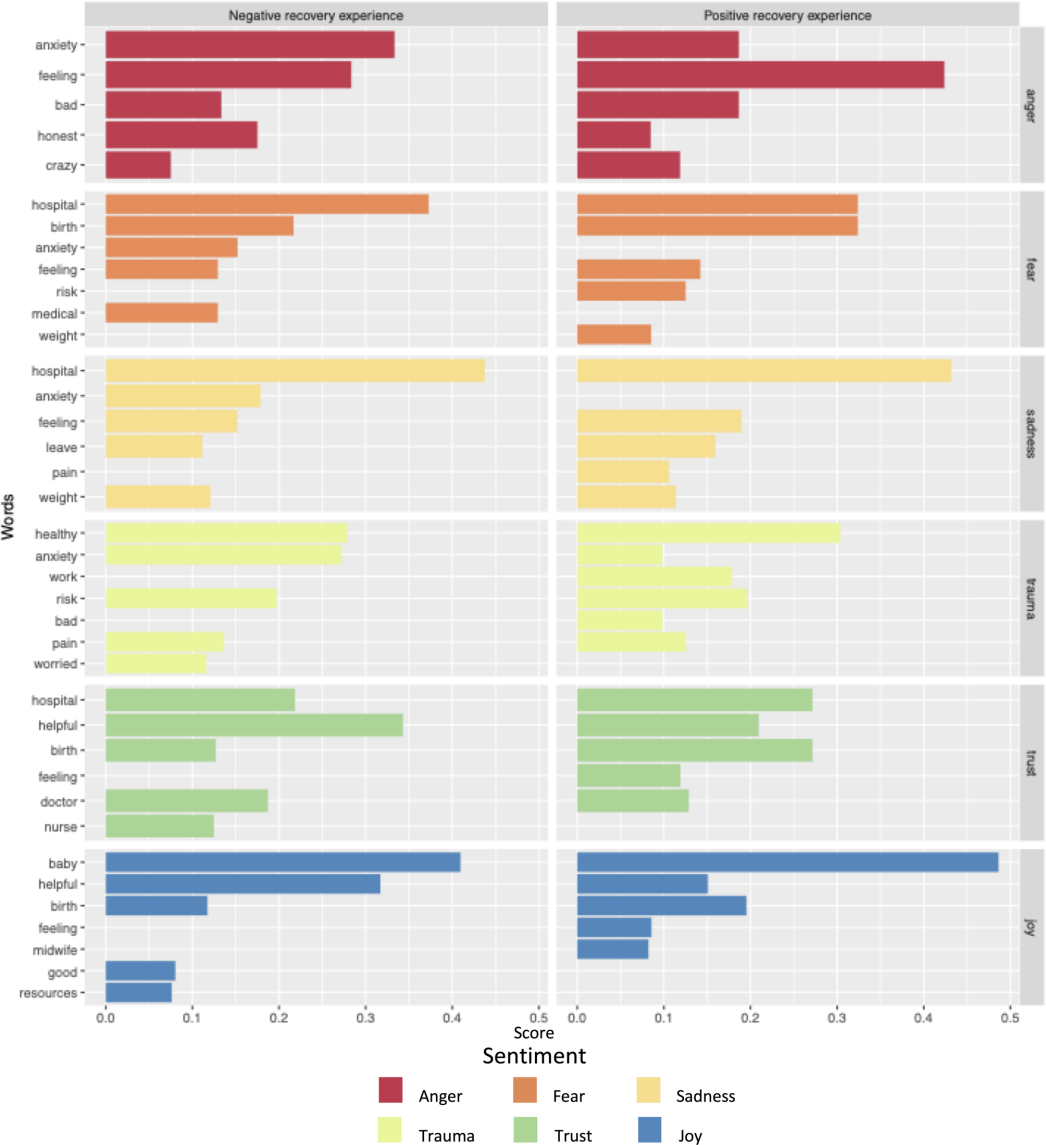
Matched terms for representative sentiment sub-lexicons in positively and negatively framed postpartum recovery experiences. Each matched term’s score is a percentage of the terms matched within that sentiment that matched with respect to the given term

As demonstrated in Fig. 4, the difference in *trust* score between positively and negatively framed postpartum experiences is largely attributed to the word “helpful”. Qualitative review of the use of the work “helpful” in negatively framed postpartum experiences talk demonstrated that it was used to express wants or needs individuals had in their recovery process or for hypothetical future postpartum experiences:

“So I was dismissed on my, how I was feeling, so having had that like, support group of people, like, I hear you. I, I know what you're going through, you know, I, I see you, would've been helpful for me cause I felt like - I was dealing with it alone.”

“The other thing that I think would be interesting or helpful is to like have a peer - Program. So like, somebody who like experienced preeclampsia or hypertension or whatever and has been trained to be a peer support - could be helpful.”

Uses of “helpful” referred to a variety of supports and services ranging from mood support to visiting nurse care– when specifically discussing HDP-related issues, participants highlighted the need for emotional and informational support, especially from peers. This was referenced both in terms of formal support groups and less formal interactions that lessened feelings of isolation:

“Just not feeling alone and, uh, I actually, there's a girl who lives real close to me. And she ended up having a very similar postpartum preeclampsia experience. Um, and so being able to connect with her and, like, commiserate with somebody who went through what I went through was really, really helpful.”

Positively framed speech had fewer mentions of “anxiety” across the emotive sub-lexicons containing the term “anxiety”. The sub-lexicons containing mentions of “anxiety” also had relatively high numbers of “baby” and “birth” mentions, even in talk identified as being about the postpartum experience. Uses of “feeling” were also disproportionately skewed towards positively framed speech.

### Topics and themes identified across care contexts

**Fig. 5:**
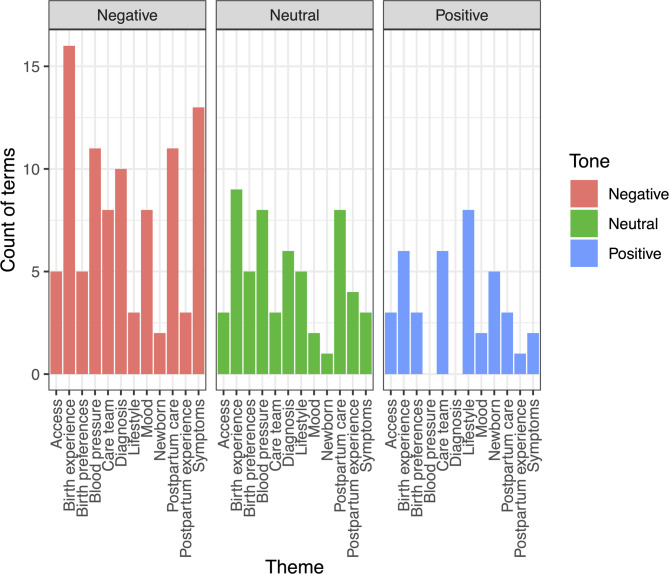
Matched-term frequency for all negative emotion sub-lexicons by qualitatively identified theme within negative birth and postpartum recovery experience talk. Terms are grouped by qualitatively assessed tone for the turns at talk in which they occurred

Speech correlating with negative emotion-sublexicon terms was grouped into the following themes: Access (e.g., ability to secure an appointment or receive a provider callback), Birth Experience (e.g., length of induction), Birth Preferences (e.g., discussion of an a priori plan to have a doula present), Blood Pressure (e.g., talk about the process of taking a blood pressure or management of a given reading), Care Team (e.g., specific comments on a nurse or provider), Diagnosis (e.g., how one was told they had an HDP), Lifestyle (e.g., discussion of diet and exercise), Mood (e.g., experience with depression or seeking therapy), Newborn (e.g., issues pertaining to a baby), Postpartum Care (e.g., discussion of appointment cadence preferences), Postpartum Experience (e.g., narrating the events occurring in a given appointment), and Symptoms (e.g., descriptions of a headache). Within the negative emotion sub-lexicons of *trauma, anger, fear,* and *sadness*, the majority of lexicon matches were in the setting of turns with a negative tone (see Fig. 5). In addition to talk pertaining to “Birth experience” (reviewed above in Birth experiences with hypertensive disorders), the topics of Blood pressure, Symptoms, Postpartum Care, and Diagnosis contained the vast majority of lexicon matches within turns with a negative tone.

Blood pressure monitoring was often accompanied by concern among patients with HDPs, stemming from unclear communication about how the readings would inform their care.

“I take my blood pressure so often, um, and it's just like not always clear what everyone's going to do with the information or like what happens when certain things show up like, monitoring for the sake of monitoring has been driving me a little crazy.”

“I'm fine with monitoring and I'm fine with communication. I just feel like I need to know what it's for, because it's been a lot of like, track this and tell us what's happening and then we're either going to do nothing or like tell you you have to randomly go to the hospital.”

More neutral sub-lexicon terms, such as “feeling,” were used negatively in the context of blood pressure monitoring. Participants shared anecdotes of attempting to assert agency through choosing the conditions under which they measured their blood pressure. This was especially true when favorable readings could influence clinical decisions, such as avoiding admission or discharge:

“I said would you please quit bringing the cuff in cause I might get, you know, I'm like oh, my God, this is going to dictate if I get to go home or not. So, I said, would you mind just leaving the machine in the room and letting me take my own BP's when I'm feeling calm and you know, and then document them and they would stay on the screen and you would see them and if I have some good readings overnight would we then be able to entertain me going home the next morning.”

Where blood pressure talk generally focused on monitoring and plans, negatively framed symptom talk pertained to the experience of having an HDP or being postpartum from a difficult vaginal birth or major abdominal surgery:

“I was really surprised by the amount of pain that I was in, and I think it was from that complication and the surgery that I had. Um, I, it was pretty, pretty bad.”

“I was, I just felt really bad across the board. Um, the upper, you know, the quadrant pain around where my liver is, which I now know, um, you know, that, that was kind of the biggest telltale sign, but definitely also nausea, a little bit of, you know, dizziness. Um, just feeling unwell.”

Whether due to overwhelming symptoms or no symptoms at all, participants generally felt disconnected to or uninformed of their medical course. Many expressed feelings of frustration and disappointment at their limited understanding of their HDP diagnosis. While this lack of clarity was attributed in part to the circumstances and intensity of birth, participants also pointed to inadequate communication from their care teams. In some cases, the lack of communication approached a sense of dehumanization, potentially impacting both recovery process and relationship with the healthcare system:

“No one was telling me what was happening to me medically. And no one was even, like, acknowledging me as a human. So it was, it was just an odd experience."

“I think it took me, uh, until after I got home to really recognize how sick I had been. I think the combination of not having pictured birth in advance and then being knocked out on the magnesium and, um, still being in the hospital, um, where I was, my retention of what was going, like, the morning before I left the hospital I remember asking the doctor can you just go over again what happened and, and confirm did I have preeclampsia?"

The theme of poor communication and insufficient education was also evident in negative postpartum care talk. Overwhelmingly, participants felt a strong need for more robust counseling on HDP, citing specific knowledge gaps with respect to long term health outcomes and how to seek care:

“Well, do I need to see a cardiologist? Do I need, you know, to see a primary care physician? Like, who do I keep getting refills from?”

“Am I going to be on blood pressure medication for the rest of my life? Like, do I just have high blood pressure now? Like is this going to go away at some point?”

“I wish someone would have sat down and said, these are your risk factors in the future.”

Negative sub-lexicon terms were used in positive talk about lifestyle modifications in particular (see Fig. 5). This was driven by the term “healthy” as part of the “trauma” sub-lexicon. In the training dataset, it was used to contrast medical goals of a “healthy” birth with the more inclusive goal-set of a positive birth experience. This was not the case in the interview speech analyzed here, where healthy was used to describe aspirational diet and exercise habits.

## Discussion

Despite the known risks to short and long-term maternal health conferred by HDPs, major gaps exist in the supports offered throughout the postpartum period for those recovering from these disorders. These gaps are best identified through the lived experiences of patients, perspectives that have been under-reported in the postpartum HDP literature. The current study interviewed a diverse sample of people recently affected by an HDP and used both qualitative and quantitative techniques to extract and interpret key themes in their narratives, finding that talk about “anxiety” and other fear-based language was a focus of those who cast their birth and postpartum experiences with HDP in a negative light.

Participants in our study characterized blood pressure monitoring and management as a negative healthcare experience, whereas birth itself had both positive and negative emotions associated with it. Even when telling birth stories that were far from a patient-centered birthing care experience [23], participants still identified blood pressure monitoring and management as the focus of negativity in healthcare interactions. While it may not be surprising that the need to have additional medical observation was perceived negatively, the participants in the study highlighted that the negative experience was not because of their diagnosis, but rather stemmed from the poor communication and support that was provided surrounding this very common pregnancy complication. This highlights the need for obstetrical care teams to set a higher standard of care around this common diagnosis. Prior studies have examined particular situations where blood pressure monitoring or management worsened patient perspectives on healthcare [13, 15], but the many ways in which the concept of blood pressure negatively impacts the experience of people recovering from HDPs has been underappreciated. Interventions are needed to address these feelings of negativity towards blood pressure monitoring and management, both in the hospital and post-discharge. Self-reporting of home blood pressures as opposed to inpatient blood pressure monitoring (where clinically appropriate) is one such intervention- it has been shown to attenuate anxiety related to blood pressure [24]. This practical solution does not entirely resolve the issue of blood-pressure-related negativity though, as participants in our study with access to self-reporting still expressed negative emotions regarding the process and experience of monitoring their blood pressures.

Across birth and postpartum contexts, concern about blood pressure and its potential impact on outcomes and care was a shared theme. Participants identified a lack of communication, control over the clinical interpretation of their blood pressures, and inclusion in their own care as key sources of distress and anxiety surrounding their HDP diagnosis. This reflects the theme of fear and uncertainty [25] as well as fragmented information provision [26] identified in smaller scale interview studies of people who were postpartum from a pregnancy complicated by an HDP. It is also congruent with prior research showing a low baseline knowledge of HDPs among pregnant people that is modifiable with education from providers [27], counseling that is often omitted. Structured antenatal education on HDPs has been shown to increase patient knowledge [28], but anxiety has not been consistently evaluated as an outcome of trialed educational interventions. Participants repeatedly identified additional education and peer support as needs that would have positively impacted their recovery process and potentially decreased their anxiety. The association between these care gaps and the language of anxiety is an incremental contribution of the present work, one that suggests improving patient-provider communication in this area may be an opportunity to support patients’ mental health, where those postpartum from a pregnancy complicated by an HDP are at higher risk for mood disorders [28, 29]. Improved communication is a necessary first step to avoid the dehumanization experienced by many of the participants in this cohort.

Notably, however, previously trialed care models that have involved multiple healthcare touchpoints in the postpartum period have reported poor attendance [30]. Though participants in our study were open to and largely positive about the potential for structured lifestyle interventions, they emphasized the importance of access to a community of people who had experienced HDPs as a key aspect to recovery. The provision of emotional support from a trusted circle has been previously recognized as a need in the recovery process from an HDP [28], but the specific want for a community of peers who have been through similar experiences has received relatively little attention. Furthermore, it points to opportunities for integration of peers into the provision of postpartum care for those affected by HDPs via group care models [31] or medically organized support groups.

The strengths of this study include its size and its incorporation of quantitative methods (e.g., sentiment analysis) to provide novel insights on patient experiences with and needs for HDP recovery. We present the largest postpartum HDP interview study to date [14], with geographic and experiential diversity represented in our sample. While smaller, more focused studies provide considerable depth in their explorations of HDP experiences, our quantitative methods were able to extract key terms and topics for qualitative review, allowing for comparable depth of analysis despite large amounts of recorded speech. Such approaches are innovative in the literature on patient experiences with HDPs and necessary to generate generalizable findings that can form the basis for large-scale initiatives designed to support this population.

This study was limited by its lack of interview-matched demographic data, its under-representation of groups at high risk of HDP-related complications, and its reliance on patient reported medical information. The study design decision not to link a given interviewee’s survey and interview data constrained our ability to compare responses across demographics. From the survey data, we do know that the majority of respondents identified as White, but we are unable to associate interviews to Black or Hispanic identifying individuals in order to isolate their feedback. Future work would ideally allow for analysis specific to demographic subgroups, especially those known to have elevated risk of HDPs and HDP related complications. We did not assess details of delivery location, such as zip code or practice type (e.g., academic, community), which may have provided additional insight into differences in patient experience. Additionally, we required that all participants speak English which further limited greater cultural diversity. We also collected only patient-reported data. Though this was consistent with the stated goal of the study, medical information such as gestational age at delivery was not confirmed by a review of the medical record. Lastly, due to our method of recruitment, there is risk of selection bias as those with negative experiences may be more likely to report them.

We aimed to better understand patients’ experiences in recovery from an HDP and identify possible targets for care advancement in a large, broadly recruited sample of recently pregnant people. Experiences with blood pressure measurement and associated management were negatively framed as anxiety-inducing by our participants. Our analyses of their speech identified anticipatory guidance surrounding HDPs, communication about blood pressure management, and long-term risk education as areas of opportunity to improve care. Furthermore, participants expressed a need for interaction with peers who have experienced HDPs. Further stratification of demographic data and exploration of the impact of cesarean versus vaginal birth on the experiences of individuals diagnosed with HDPs represent important areas for ongoing research. Future interventions to support patients recovering from HDPs should consider integrating peer support and iterative education about HDPs into programs designed to reduce the lifelong sequelae of these disorders.

## Supplementary Information

Additional file 1. Supplemental table 1. Supplemental table 2.

Additional file 2. List of Appendices.

## Data Availability

De-identified interview data and a full list of the 14,000 + lexicon items used in the sentiment analysis are available from the authorship team by written request to the corresponding author.

## Abbreviations

CHIP: Children's Health Insurance Program
HDP: Hypertensive Disorders of Pregnancy
HEARD: Hypertension Experiences Affecting Recovery from Delivery
HELLP: Hemolysis Elevated Liver Enzymes Low Platelet
NICU: Neonatal Intensive Care Unit
SW: Social Worker
US: United States

## References

[1] Ford ND, Cox S, Ko JY, et al. Hypertensive disorders in pregnancy and mortality at delivery hospitalization — United States, 2017–2019. MMWR Morb Mortal Wkly Rep. 2022;71:585–91. 10.15585/mmwr.mm7117a1. 35482575 10.15585/mmwr.mm7117a1 PMC9098235

[2] Ghosh G, Grewal J, Männistö T, et al. Racial/ethnic differences in pregnancy-related hypertensive disease in nulliparous women. Ethn Dis. 2014;24(3):283–9. 25065068 PMC4171100

[3] Khosla K, Heimberger S, Nieman KM, et al. Long-term cardiovascular disease risk in women after hypertensive disorders of pregnancy: recent advances in hypertension. Hypertension. 2021;78(4):927–35. 10.1161/HYPERTENSIONAHA.121.16506. 34397272 10.1161/HYPERTENSIONAHA.121.16506 PMC8678921

[4] Ying W, Catov JM, Ouyang P. Hypertensive disorders of pregnancy and future maternal cardiovascular risk. J Am Heart Assoc. 2018;7(17):e009382. 10.1161/JAHA.118.009382. 30371154 10.1161/JAHA.118.009382 PMC6201430

[5] Lovgren T, Connealy B, Yao R, Dahlke JD. Postpartum management of hypertension and effect on readmission rates. Am J Obstet Gynecol MFM. 2022;4(1):100517. 10.1016/j.ajogmf.2021.100517. 34757235 10.1016/j.ajogmf.2021.100517

[6] Raina J, El-Messidi A, Badeghiesh A, Tulandi T, Nguyen TV, Suarthana E. Pregnancy hypertension and its association with maternal anxiety and mood disorders: a population-based study of 9 million pregnancies. J Affect Disord. 2021;281:533–8. 10.1016/j.jad.2020.10.058. 33388464 10.1016/j.jad.2020.10.058

[7] Kountanis JA, Muzik M, Chang T, Langen E, Cassidy R, Mashour GA, Bauer ME. Relationship between postpartum mood disorder and birth experience: a prospective observational study. Int J Obstet Anesth. 2020;44:90–9. 10.1016/j.ijoa.2020.07.008. 32861082 10.1016/j.ijoa.2020.07.008

[8] Stuebe A, Auguste T, Gulati M. Optimizing postpartum care. Obstet Gynecol. 2018;131(5):E140–50. 29683911 10.1097/AOG.0000000000002633

[9] Triebwasser JE, Janssen MK, Sehdev HM. Postpartum counseling in women with hypertensive disorders of pregnancy. Am J Obstet Gynecol MFM. 2021;3(1):100285. 10.1016/j.ajogmf.2020.100285. 33451593 10.1016/j.ajogmf.2020.100285

[10] Lanier JB, Bury DC, Richardson SW. Diet and physical activity for cardiovascular disease prevention. Am Fam Physician. 2016;93(11):919–24 (**PMID: 27281836**). 27281836

[11] Sawan MA, Calhoun AE, Fatade YA, Wenger NK. Cardiac rehabilitation in women, challenges and opportunities. Prog Cardiovasc Dis. 2022;70:111–8. 10.1016/j.pcad.2022.01.007. 35150655 10.1016/j.pcad.2022.01.007

[12] Li J, Zhou Q, Wang Y, et al. Risk factors associated with attendance at postpartum blood pressure follow-up visit in discharged patients with hypertensive disorders of pregnancy. BMC Pregnancy Childbirth. 2023;23:485. 10.1186/s12884-023-05780-6. 37391694 10.1186/s12884-023-05780-6 PMC10311897

[13] Thomas NA, Drewry A, Racine Passmore S, Assad N, Hoppe KK. Patient perceptions, opinions and satisfaction of telehealth with remote blood pressure monitoring postpartum. BMC Pregnancy Childbirth. 2021;21:153. 33607957 10.1186/s12884-021-03632-9 PMC7896378

[14] Sakurai S, Shishido E, Horiuchi S. Experiences of women with hypertensive disorders of pregnancy: a scoping review. BMC Pregnancy Childbirth. 2022;22(1):146. 10.1186/s12884-022-04463-y. 35193516 10.1186/s12884-022-04463-y PMC8864783

[15] Viswanathan R, Little SE, Wilkins-Haug L, et al. The patient experience of a postpartum readmission for hypertension: a qualitative study. BMC Pregnancy Childbirth. 2024;24:358. 10.1186/s12884-024-06564-2. 38745136 10.1186/s12884-024-06564-2 PMC11094995

[16] Mohammed, S. M. (2021). Sentiment analysis: Automatically detecting valence, emotions, and other affectual states from text. In Emotion measurement (pp. 323–379). Woodhead Publishing.

[17] Austin MA, Saxena A, O’Malley TJ, Maynes EJ, Moncure H, Ott N, et al. Computational sentiment analysis of an online left ventricular assist device support forum: positivity predominates. Ann Cardiothorac Surg. 2021;10(3):375. 34159118 10.21037/acs-2020-cfmcs-fs-11 PMC8185377

[18] Cero I, Luo J, Falligant JM. Lexicon-based sentiment analysis in behavioral research. Perspect Behav Sci. 2024. 10.1007/s40614-023-00394-x. 38660506 10.1007/s40614-023-00394-x PMC11035532

[19] Shankar R, Xu Q, Bundele A. Patient voices in dialysis care: sentiment analysis and topic modeling study of social media discourse. J Med Internet Res. 2025;27:e70128. 40372782 10.2196/70128 PMC12123232

[20] Taboada M. Sentiment analysis: an overview from linguistics. Annu Rev Linguist. 2016;2(1):325–47.

[21] Tang J, Arvind V, White CA, Dominy C, Kim JS, Cho SK, Walsh A. Using sentiment analysis to understand what patients are saying about hand surgeons online. Hand. 2023;18(5):854–60. 34969297 10.1177/15589447211060439 PMC10336809

[22] Czarnek G, Stillwell D. Two is better than one: Using a single emotion lexicon can lead to unreliable conclusions. PLoS ONE. 2022;17(10):e0275910. 10.1371/journal.pone.0275910. 36240202 10.1371/journal.pone.0275910 PMC9565755

[23] Leinweber J, Fontein-Kuipers Y, Karlsdottir SI, Ekström-Bergström A, Nilsson C, Stramrood C, Thomson G. Developing a woman-centered, inclusive definition of positive childbirth experiences: a discussion paper. Birth. 2023;50(2):362–83. 35790019 10.1111/birt.12666

[24] Yeh PT, Rhee DK, Kennedy CE, Zera CA, Lucido B, Tunçalp Ö, de Gomez Ponce Leon R, Narasimhan M. Self-monitoring of blood pressure among women with hypertensive disorders of pregnancy: a systematic review. BMC Pregnancy Childbirth. 2022;22(1):454. 35641913 10.1186/s12884-022-04751-7 PMC9152837

[25] Arntzen E, Jøsendal R, Sandsæter HL, Horn J. Postpartum follow-up of women with preeclampsia: facilitators and barriers—a qualitative study. BMC Pregnancy Childbirth. 2023;23(1):833. 38049716 10.1186/s12884-023-06146-8 PMC10694896

[26] Andersson ME, Rubertsson C, Hansson SR. The experience of provided information and care during pregnancy and postpartum when diagnosed with preeclampsia: a qualitative study. Eur J Midwifery. 2021. 10.18332/ejm/139488. 34568778 10.18332/ejm/139488 PMC8424697

[27] You WB, Wolf M, Bailey SC, Pandit AU, Waite KR, Sobel RM, Grobman W. Factors associated with patient understanding of preeclampsia. Hypertens Pregnancy. 2012;31(3):341–9. 20860492 10.3109/10641955.2010.507851

[28] You WB, Wolf MS, Bailey SC, Grobman WA. Improving patient understanding of preeclampsia: a randomized controlled trial. Am J Obstet Gynecol. 2012;206(5):431-e1. 10.1016/j.ajog.2012.03.006 22542120

[29] Roberts LM, Davis GK, Homer CS. Pregnancy with gestational hypertension or preeclampsia: a qualitative exploration of women’s experiences. Midwifery. 2017;46:17–23. 28110162 10.1016/j.midw.2017.01.004

[30] Roberts L, Henry A, Harvey SB, Homer CS, Davis GK. Depression, anxiety and posttraumatic stress disorder six months following preeclampsia and normotensive pregnancy: a P4 study. BMC Pregnancy Childbirth. 2022;22(1):108. 35130869 10.1186/s12884-022-04439-y PMC8822717

[31] Gladstone RA, Pudwell J, Pal RS, Smith GN. Referral to cardiology following postpartum cardiovascular risk screening at the maternal health clinic in Kingston, Ontario. Can J Cardiol. 2019;35(6):761–9. 10.1016/j.cjca.2019.03.008. 31151712 10.1016/j.cjca.2019.03.008

