# Supplementary material for: Hypertension Experiences Affecting Recovery from Delivery (HEARD): a mixed-methods interview study of postpartum people affected by hypertensive disorders of pregnancy
Source: BMC Pregnancy Childbirth. 2025 Sep 30;25:954. doi: 10.1186/s12884-025-08013-0 (PMC12487555; doi:10.1186/s12884-025-08013-0)
Supplement: Supplementary file 1 — Additional file 1. Supplemental table 1. Supplemental table 2. [file 12884_2025_8013_MOESM1_ESM.docx]

**Supplemental table 1.**

| **Patient-reported data** | |
| --- | --- |
| First Birth | Yes  No |
| Gestational age | Pre-term  Term |
| Difficulty with blood pressure cuff | Yes  No  Not asked  Unsure |
| Discharged on blood pressure medication | Yes  No  Not asked  Unsure |
| Given instructions on monitoring of home blood pressures and symptoms | Yes  No  Not asked  Unsure |
| Satisfied with blood pressure monitoring instructions | Yes  No  Not asked  Unsure |
| Satisfied with blood pressure follow up | Yes  No  Not asked  Unsure |
| Counseled on lifelong risks of HDP diagnosis | Yes  No  Not asked |
| Cardiology follow up | Yes  No  Not asked |
| Primary support people at home | *open ended* |
| Satisfied with maternity leave length | Yes  No  Not asked |
| Lactation difficulties | Yes  No  Not asked |
| Desire for lactation support | Yes - in person  Yes - virtual  No  Not asked |
| Desire for new patient support group | Yes - in person  Yes - virtual  No  Not asked |
| Access to postpartum doula | Yes  No  Not asked |
| Desire for newborn education | Yes - in person  Yes - virtual  No  Not asked |
| Communication with postpartum provider | Visit before postpartum visit  No contact until postpartum visit  Communicated virtually |
| Example provided of poor communication | Yes  No  Not asked |
| Example provided of good communication | Yes  No  Not asked |
| Postpartum exercise habits | *open ended response recorded* |
| Desired postpartum exercise support | *open ended response recorded* |
| Pre-pregnancy exercise habits | *open ended response recorded* |
| Postpartum diet | *open ended response recorded* |
| Desired postpartum diet support | *open ended response recorded* |
| Pre-pregnancy dietary habits | *open ended response recorded* |
| Pre-existing mood symptoms | Yes  No  Not asked  Patient unsure |
| Peripartum mood diagnosis | Yes  No  Not asked  Unsure |
| Mood support received | *open ended response recorded* |
| Counseling for diagnosis | Adequate  Inadequate  Not asked  Unsure |
| Counseling for future pregnancy | Adequate  Inadequate  Not asked  Unsure |
| Preferred source of information | *open ended response recorded* |
| Preferred emotional support source | *open ended response recorded* |
| **Subjective assessed data** |  |
| Birth experience | Positive  Neutral  Negative  Not asked |
| Experience with hospital care team | Positive  Neutral  Negative  Not asked |
| Recovery experience | Positive  Neutral  Negative  Not asked |

**Supplemental table 2.**

| **Linguistic term** | **Explanation** |
| --- | --- |
| Talk, speech | Transcribed speech from the interviews. |
| Sub-lexicons | Groupings of related words within a broader category.  *Example: in a lexicon of furniture terms, “couch”, “sofa”, “bench” would be a sub-lexicon of seating for multiple people* |
| Turns at talk (i.e., “turns”) | Back and forth of conversation, where each speaker’s speech prior to ceding the floor is a turn.  Example:  *Speaker A, turn 1: Hi how are you?*  *Speaker B, turn 1: I’ve been fine.*  *Speaker A, turn 2: The weather has been nice. Hoping to toke a trip this weekend.*  *Speaker B, turn 2: Oh really? I just got back from vacation.* |
| Polarity | Positive or negative forms of a word, phrase, or concept that can be altered by positive/ negative polarity items.  *Example:*  *Positive polarity of the word “pretty”: That dress is pretty*  *Negative polarity of the word “pretty”: That dress is not pretty*  *Negative polarity of the phrase containing “pretty” changing the polarity of “pretty”: It’s not that the dress is pretty* |
| Emotive valence | Emotive valence- the conveyance of positively or negatively framed subjective feelings.  Example:  *Negative example: It makes me so mad when Joe does that.*  *Positive example: I absolutely love the new director.* |
| Relational valence | Affective value (positive or negative) assigned to a thing, event, or person determined by its relationship to another entity or context, rather than as a fixed value.  *Positive example: Jim is a friend of Louise.*  *Negative example: That salesperson was mean to me.* |
