# Supplementary material for: Hypertension Experiences Affecting Recovery from Delivery (HEARD): a mixed-methods interview study of postpartum people affected by hypertensive disorders of pregnancy
Source: BMC Pregnancy Childbirth. 2025 Sep 30;25:954. doi: 10.1186/s12884-025-08013-0 (PMC12487555; doi:10.1186/s12884-025-08013-0)
Supplement: Supplementary file 2 — Additional file 2. List of Appendices. [file 12884_2025_8013_MOESM2_ESM.docx]

**List of Appendices**

**Appendix A: Screening Questionnaire**

A.1 Introduction

A.2 Screening Questions

A.3 Qualifications

A.4 Additional Questions

A.5 Outro

**Appendix B: Interview Script**

B.1 Introduction

B.2 Obstetric History

B.3 Birth/HDP Experience

B.4 Postpartum Experience

B.5 Extra Questions

B.6 Outro

**Appendix A: Screening Questionnaire**

A.1 Introduction

Thank you for your interest in the Hypertension Experiences Affecting Recovery from Delivery (HEARD) study! Please answer the following questions to see if you qualify to participate.

A.2 Screening Questions

1. What is your age?
2. How many months ago did you give birth?
3. Which of the following conditions affected your pregnancy or postpartum time?
   1. High blood pressure (present before you were 20 weeks pregnant)
   2. High blood pressure in pregnancy (also known as gestational hypertension, new during pregnancy or after delivery)
   3. Preeclampsia (high blood pressure in pregnancy with lab changes or symptoms of high blood pressure)
   4. Eclampsia (seizures from high blood pressure)
   5. HELLP (changes in kidney, liver, or blood labs)
   6. None of the above

A.3 Qualification

Congratulations, you qualify for the HEARD study!

We would like to know a little more about you. Please answer the following questions to help us learn more about people with high blood pressure problems in pregnancy.

After you answer these questions, you will be directed to a scheduling website to choose a time for your interview. Once you have completed your interview, you will be sent your $50 Amazon gift card.

1. Would you like to continue?
2. Yes
3. No

A.4 Additional Questions

1. How would you describe your race and ethnicity? [Select all that apply]
   1. American Indian or Alaska Native
   2. Asian
   3. Black or African American
   4. Hispanic or Latino
   5. Native Hawaiian or Other Pacific Islander
   6. White
   7. Prefer not to say
2. How many weeks pregnant were you when you gave birth (ex. 38 weeks)?
3. Was this your first birth?
   1. Yes
   2. No
4. How many family members, including yourself, do you currently live with?
   1. One or two
   2. Between 3 and 5
   3. More than 5
5. What is your housing situation today?
   1. I have housing
   2. I do not have housing (staying with others, in a hotel, in a shelter, living outside on the street, on a beach, in a car, or in a park)
   3. I choose not to answer this question
6. Are you worried about losing your housing?
   1. Yes
   2. No
   3. I choose not to answer this question
7. What is the highest level of school that you have finished?
   1. Less than high school degree
   2. High school diploma or GED
   3. More than high school
   4. I choose not to answer
8. What is your current work situation?
   1. Unemployed
   2. Part-time or temporary work
   3. Full-time work
   4. Otherwise unemployed but not seeking work (ex: student, retired, disabled, unpaid primary caregiver)
9. Insurance What is your main health insurance?
   1. None/Uninsured Medicaid
   2. CHIP Medicaid Medicare
   3. Other Public
   4. Insurance (not CHIP)
   5. Other Public Insurance (CHIP)
   6. Private Insurance
10. In the past year, have you or any family members you live with been unable to get any of the following when it was really needed? Check all that apply.
    1. Food
    2. Utilities
    3. Medicine or Any Health Care (Medical, Dental, Mental Health, Vision)
    4. Phone
    5. Clothing
    6. Child care
    7. I choose not to answer this question
11. Has lack of transportation kept you from medical appointments, meetings, work, or from getting things needed for daily living? Check all that apply.
    1. Yes, it has kept me from medical appointments or from getting medication
    2. Yes, it has kept me from non-medical meetings, appointments, work, or from getting things that I need
    3. No
    4. I choose not to answer this question
12. How often do you see or talk to people that you care about and feel close to? (For example: talking to friends on the phone, visiting friends or family, going to church or club meetings)
    1. Less than once a week
    2. 1 or 2 times a week
    3. 3 to 5 times a week
    4. 5 or more times a week
    5. I choose not to answer this question
13. Stress is when someone feels tense, nervous, anxious, or can’t sleep at night because their mind is troubled. How stressed are you?
    1. Not at all
    2. A little bit
    3. Quite a bit
    4. Somewhat
    5. Very much
    6. I choose not to answer this question

A.5 Outro

You are now ready to schedule an interview. Please read the following privacy statement:

The interviews will be conducted by a trained moderator. There may be another researcher present for quality assurance purposes. The interview will be audio recorded for notetaking and analysis. The healthcare communication company Verilogue, Inc. will be securely storing the interview recording for these purposes. You can read more about their privacy policies here. Please note that the data you provide will be stored in an anonymized format separately from the contact information you use to schedule your interview and receive compensation. The researchers, moderators, and Verilogue, Inc. will NOT have access to any data other than that which you choose to provide during the scheduling process and interview. You are strongly encouraged to AVOID giving personal details such as your full name or address. You may use initials or a pseudonym during the interview to further protect your privacy if desired.

If you would like to proceed with the interview scheduling process, please click the link below to schedule. When signing up for a time, please use your first and last INITIALS ONLY to protect your privacy.

As a reminder, you will receive $50 in compensation for your time spent completing the interview. Thank you for your interest in the HEARD study- we look forward to talking with you!

**Appendix B: Interview Script**

B.1 Introduction

Thank you for agreeing to participate today. My name is [moderator first name] and I am a [role]. You don’t have to use your real first name, you can tell me any name you wish, but how should I call you?

Thank you for agreeing to participate today. My name is [moderator first name] and I am a [role]. Just as a reminder, today’s discussion is about your recent pregnancy, birth, and postpartum experience. Please feel free to be open throughout our discussion — there are no right or wrong answers. Your participation in this research is entirely voluntary so if you feel uncomfortable at any point or need to pause, just let me know.

We would like to record this discussion so the team can review our conversation at a later date. Any details or experiences you share today will be kept confidential, with only members of the research team having access to the recording. Your contact information will not be connected to the recording. Do I have your permission to record? This conversation may take up to an hour of your time. You will be paid for your time.

As you know, the reason you were invited to participate is because you had issues with high blood pressure in pregnancy. As you might have discussed with your doctor or midwife, having higher blood pressures in pregnancy puts you at risk for having high blood pressure later in life. People with high blood pressure in pregnancy also have more heart attacks and strokes as they age. Making lifestyle changes with diet and exercise may help decrease the risk of these events. We are hoping to develop a program to help people make these changes soon after birth. We want the program to provide emotional support as well, because having blood pressure issues in pregnancy can be quite stressful too. Before we get too far into that conversation though, we would love to hear about your birth experiences.

B.2 Obstetric History

- Were you diagnosed with preeclampsia, gestational hypertension, or HELLP syndrome?
- At what point in your pregnancy were you diagnosed? (clarify weeks, before or after delivery)
- How far along were you when you delivered (specify weeks)?
- Did you receive Magnesium at any point in your pregnancy or postpartum? This is a medicine that would have been given to you in your IV.
  - At what point did they start Magnesium
- Did your baby need to stay in the NICU? Are you sharing why and what that was like?
- How long did you stay in the hospital after giving birth? Can you tell me a bit about what that was like?

B.3 Birth/HDP Experience

Please tell us whatever you're comfortable sharing about your birth experience.

Prompts:

- What were you hoping for in your birth experience?
- What were your concerns? Worries?
- If you were induced, what is your understanding of why induction was recommended?
- Tell me more about what labor was like for you.
- How did your blood pressure issues affect your labor and birth?
- What was your postpartum time like in the hospital?
- What instructions were you given for monitoring your blood pressure at home?
  - What was it like measuring your blood pressure at home?
  - Did you have any difficulties with your cuff?
- What were you told to look out for at home?

B.4 Postpartum Experience

Thank you. Can you tell me about what life was like at home once you left the hospital?

Prompts:

- What would have made your postpartum experience better in the first few days? How about the first few weeks? How about the first few months? (if applicable)
- How did your diet habits change from pregnancy? How about from before you were pregnant?
- How did your exercise habits change from pregnancy? How about from before you were pregnant?
- Were your diet and exercise habits consistent with your goals?
- Who supported you in your diet and exercise goals?
- What role should your doctor have in supporting your lifestyle goals?
  - Would you have been interested in additional visits with your OBGYN? Why or why not?
  - Would you have been interested in more frequent lab monitoring (e.g., lipids, A1C level, etc)? Why or why not?
- Would you have been interested in support groups on things like diet, exercise, new parent support, or postpartum mood symptoms, etc?
  - If yes, how would you prefer to meet? (virtual vs in person)?
  - What number of participants would be ideal?
    - What types of people should be included?
  - What frequency of meetings would have been appropriate?
    - Would this be different right after your baby was born (0-3 mo postpartum) vs later after the baby was born (>3 mo postpartum)?
  - If support groups were in person, would it be helpful if child care was provided in a group setting?
- Would you have been interested in additional exercise resources?
  - How would you prefer to receive that information:
    - 1:1 session with PT/exercise instructor (preference for live or virtual?)
    - Group exercise classes (preference for live or virtual?)
    - Library of pre-recorded exercise videos
    - Subscription to 3rd party home exercise program (peloton subscription, online yoga, etc)
    - Written communications (e.g., email, newsletter)
- Would you have been interested in additional nutrition resources?
  - How would you prefer to receive that information:
    - 1:1 session with a nutritionist (preference for live or virtual?)
    - Group sessions with nutritionists (preference for live or virtual?)
    - Pre-recorded video content on healthy eating
    - Subscription to 3rd party meal-planning company (blue apron, hello fresh, etc)
    - Written communications (e.g., email, newsletter)
- Would you have been interested in more frequent touchpoints with hospital social workers? Further, more long term support both emotionally and socially.
- Anything that you thought that would be helpful after birth that you did not receive?
- Did you struggle with mood or anxiety after birth?
  - Would you have been interested in more frequent check ups on your mood by members of your care team?
    - If yes, what format of communication would be best: call, text, app based messaging, virtual appointments
    - If yes, who would you prefer to interact with: registered SW, nursing, etc.
  - Would you have been interested in a referral to a psychiatrist or a member of our peripartum mood clinic?
  - Would you have been interested in a support group of parents who are currently or have previously struggled with postpartum mood concerns?
    - Zoom, 1 on 1, message board
- Is there anything else you’d like to share with us about how you would like to be supported postpartum?

B.5 Extra Questions

- What would you have wanted to know about [DISEASE THEY HAD] prior to your diagnosis?
  - If you could go back and talk to yourself prior to being diagnosed with [DISEASE THEY HAD] what would you tell yourself?
  - What would you have wanted to know about the birth or postpartum process?
- What terms do you identify with?
  - “high risk”
  - “[DISEASE THEY HAD] survivor”
  - “history of [DISEASE THEY HAD]”
- How has having [DISEASE THEY HAD] affected your planning for future pregnancies?
  - If not planning a future pregnancy, did your blood pressure problems affect this choice?
  - I hope you have the opportunity to talk to your doctor or have a preconception visit to discuss some of these things
- Is there any time you were communicating with a provider (doctor/midwife/nurse) and you felt especially well supported? Is there a time when you felt communication could be better?
- If money was no object, how would you improve the postpartum experience?

B.6 Outro

Thank you for speaking with us today. Before we close, do you have any other comments you would like to share with us? You can email us at [EMAIL] if you have questions at a later date or would like to add anything. You can also email us to withdraw from the study at any time. Your gift card will be emailed to you, you should expect it at the end of the month.
